# Exploring the World, Minimizing Risks: Travelers’ Awareness and Risk Perception of Infectious Diseases in the Post-Pandemic Era

**DOI:** 10.3390/vaccines14060485

**Published:** 2026-05-29

**Authors:** Rosa Katia Bellomo, Maria Assunta Donato, Vito Cerabona, Teresa Esposito, Alessia Perna, Giuliana Federico, Carmine Guarino, Anna Odone, Michele Sparano, Romina Sezzatini, Erika Alessandra Strangi, Eleonora Tassone, Paolo Villari, Corrado De Vito

**Affiliations:** 1 Department of Public Health and Infectious Diseases, Sapienza University of Rome, 00185 Rome, Italy; rosakatia.bellomo@uniroma1.it (R.K.B.); mariaassunta.donato@uniroma1.it (M.A.D.); giuliana.federico@uniroma1.it (G.F.); michelesparano1@gmail.com (M.S.); paolo.villari@uniroma1.it (P.V.); corrado.devito@uniroma1.it (C.D.V.); 2 Italian Ministry of Health–USMAF/SASN–Lazio, Marche, Umbria and Sardegna Regions, 00144 Rome, Italy; t.esposito@sanita.it (T.E.); a.perna-esterno@sanita.it (A.P.); carmineguarino.md@gmail.com (C.G.); r.sezzatini@sanita.it (R.S.); 3 Department of Public Health, Experimental and Forensic Medicine, University of Pavia, 27100 Pavia, Italy; anna.odone@unipv.it (A.O.); eleonora.tassone01@universitadipavia.it (E.T.); 4 Department of Prevention, ASL Roma 1, 00187 Rome, Italy; dottoressa.strangi@gmail.com

**Keywords:** travel medicine, risk perception, vaccine uptake, international travelers, pre-travel consultation

## Abstract

**Background:** Epidemiological alerts about the possible spread of different pathogens have highlighted the risk of international travelers contracting infectious diseases when visiting endemic areas. The role of travelers in disease transmission underscores the importance of pre-travel consultations, which provide critical information on health risks, vaccinations, and preventive measures. Understanding travelers’ risk perceptions and behaviors is essential for enhancing global health security in the post-pandemic era. **Methods:** A cross-sectional study (June 2023–January 2024) was conducted by administering an anonymous questionnaire at the Rome-Fiumicino Airport International Prophylaxis Clinic (USMAF-SASN). The questionnaire explored demographics, travel patterns, risk perceptions, vaccination behaviors, and sources of health information. Descriptive statistics and a multivariable logistic regression analysis were performed to identify low-risk perception predictors. **Results:** Among 217 participants, 89.8% were Italian, with a balanced representation of genders. The primary purpose of travel was tourism (61.6%), followed by work-related trip (23.1%). While 77.1% rated preventive measures as effective, 23.2% evaluated infection risk as low. Being male (aOR 3.63, 95% CI 1.37–9.61), and being a hotel user (aOR 6.27, 95% CI 2.43–16.15), was significantly associated with a lower risk perception. As expected, healthcare professionals and individuals using institutional healthcare sources showed a higher risk awareness. Vaccination uptake at the Airport Clinic was motivated by self-protection, vaccine confidence, and poor time flexibility to access local vaccination services, and last-minute plans, making the airport a more convenient option. **Conclusions:** Travelers’ risk perception is influenced by gender, profession, accommodation type, and information sources. Public health strategies should enhance health literacy, promote pre-travel consultations, and improve access to preventive services. Strengthening collaborations between health authorities, educational institutions, and the travel sector is key to mitigating health risks and ensuring global health security. Future interventions should address structural vaccination barriers and improve outreach to under-informed travelers.

## 1. Introduction

In recent years, international travel has increased, impacting the geographic spread of health threats such as Ebola virus, Zika virus, and antimicrobial-resistant pathogens [1,2,3,4]. Between 6% and 87% of travelers report illness during or after their journeys. Another study indicates that between 43% and 79% of those traveling to developing countries experience health issues, primarily traveler’s diarrhea [5]. Furthermore, a recent meta-analysis found that approximately 36% of travelers experienced diarrhea during trips lasting less than 100 days [6].

Despite this, research has consistently shown over time that travelers’ awareness and perception of the risks associated with infectious diseases are often inadequate or inappropriate given the actual risks [7,8,9]. Studies conducted on different populations, such as tourists, business travelers, visitors to friends and relatives (VFRs), students and older adults, reveal significant gaps in knowledge, attitudes and practices regarding disease risk and prevention [7,8,10]. Factors influencing risk perception include demographics, aim of travel, destination and source of health information [8,11]. Furthermore, low risk perception, misinformation, concerns about costs and barriers to accessibility not only lead to suboptimal uptake of vaccinations and chemoprophylaxis despite their availability but also discourage travelers from seeking or following health advice prior to travel [12,13,14].

However, there are several challenges associated with the evaluation of the risks of contracting infectious diseases while traveling. The main problems are incomplete counts of ill travelers and a lack of information on how many, and which people, are at risk at each destination. Clinical surveillance networks such as GeoSentinel and EuroTravNet capture only ill travelers who seek care at specialist clinics; consequently, the total number of travelers, including healthy ones, is unknown, which prevents the calculation of true incidence or risk [5,15,16,17,18]. The data are skewed towards more severe or unusual infections and towards regions with specialist centers, primarily Europe and North America [15,16,17]. In most cases, diagnostic tests are not conducted to diagnose the disease. Estimating the incidence of traveler infections, as well as the causative agents, is essential for providing appropriate health advice, but the current system has several data gaps. Multi-country travel and uncertainty regarding the place of exposure complicate the attribution of an infection to a specific destination [17,19]. Many illnesses are self-treated or go unreported, whilst some rare risks (such as rabies following animal bites) may be overestimated due to precautionary management [20]. Statistics on the number of passengers obtained from tourism authorities and national agencies differ due to the differences in data collection approaches. In most cases, a single data source cannot provide the exact number of visits, particularly in cases involving stopovers [21]. Furthermore, travelers are known to differ from residents in several ways, including behavioral, dietary, accommodation, and other factors, potentially leading to a different risk of infectious disease contraction compared to the local population.

### Objectives

The primary objective of this study was to identify factors associated with low perceived risk of infectious diseases among international travelers in the post-pandemic era. Secondary objectives were to describe pre-travel health awareness and risk perception, preventive behaviors, drivers/barriers of pre-exposure prophylaxis uptake, vaccination willingness and sources of health information in a real-world airport health service center.

## 2. Materials and Methods

### 2.1. Study Population

The study used a cross-sectional design following the Strengthening the Reporting of Observational Studies in Epidemiology (STROBE) model [22]. Data were collected between June 2023 and January 2024 through a web survey distributed via Google Forms to travelers departing from or transiting through Rome-Fiumicino “Leonardo da Vinci” International Airport (LIRF), specifically at the Maritime, Air and Border Health Offices (USMAF-SASN) clinic. Inclusion criteria required participants to be of legal age (18 years or older), transiting through the USMAF-SASN clinic, and able to understand one of the four languages in which the questionnaire was translated. Medical staff at the USMAF-SASN clinic informed all participants of the survey’s methods, objectives, and guarantees of anonymity. Informative posters regarding the study were also displayed at the USMAF-SASN clinic. The questionnaire was administered during routine clinical activities with informed consent, and the ethics approval applied specifically to the retrospective analysis of anonymized data.

### 2.2. Questionnaire

The questionnaire was adapted from an instrument previously used in the Italian context [7] and was presented in Italian, English, Spanish, and French to minimize potential language barriers. It included a demographic assessment to examine the sample’s social and personal characteristics, including gender, age, employment status, education level, nationality, religion, and political orientation. Additionally, it collected information on the destination, type of accommodation, trip setting, organization, equipment, and the reasons and frequency of the trips. Furthermore, it assessed travelers’ attitudes and perceptions of risk regarding major infectious diseases that could arise at their destination. Finally, the questionnaire explored their knowledge and attitudes toward relevant preventive measures, pre-travel prophylaxis, travel health and well-being.

### 2.3. Sample Size

The required sample size was determined based on a predicted prevalence of 75% for low-risk perception, as estimated from previous literature in travel medicine [23,24]. To achieve a 95% confidence level with a margin of error of ±7%, a minimum of 196 completed surveys was required. Participants were recruited using a convenience sampling approach among travelers visiting the airport clinic. The statistical analysis was performed using the statistical software STATA 18.0 (StataCorp LLC, College Station, TX, USA) and R (R version 4.0.0, http://www.r-project.org). A two-tailed p-value less than 0.05 was considered statistically significant.

### 2.4. Variables Description

The primary outcome was low risk perception, defined as a score of 3 or lower on a 0–10 Likert scale. This was assessed using the following question: ‘How would you rate the risk of contracting an infectious disease during your trip?’ (0 = ‘No risk’; 10 = ‘Very high risk’).

The following covariates were included in the model selected based on literature and background knowledge: sex, employment status, travel frequency, accommodation type, the preventive measures awareness and the sources of information on preventive measures.

Sex was a dummy with female as reference category. Employment status was categorized into ‘other’, unemployed, teacher, law enforcement or armed forces, airport worker and seafarer, healthcare professional, and student. Sources of information on preventive measures included health sources (family physicians or pediatricians) and non-health sources (travel agencies, family and friends, or online resources), with the latter serving as the reference category. The first category was the reference group to calculate odds ratios.

Furthermore, the variable “sources of information on preventive measures” derived from a question asking respondents where they obtained information regarding infectious diseases at their destination and was dichotomized into “institutional healthcare source” and “non-healthcare sources”. Participants were categorized into the “institutional healthcare source” group if they consulted a physician or USMAF clinic while those relying exclusively on embassies, the internet, travel agencies, or personal networks, were classified as the “non-health source”.

### 2.5. Statistical Analysis

Absolute and relative frequencies were calculated for categorical variables. Adjusted odds ratios (aOR) were estimated using a multivariable logistic regression model based on the strategy by Hosmer and Lemeshow [25] to identify predictors of low perception of risk. Variable selection was based on background knowledge. Model selection was performed using the Akaike Information Criterion (AIC). Goodness of fit was assessed using the Hosmer–Lemeshow test. The adjusted odds ratio (aOR) and the corresponding 95% confidence interval were calculated for each variable. We performed a complete case analysis. Since the proportions of missing data for incomplete variables were small (Tables 1–3), the application of missing data methods was not necessary.

To assess the model’s predictive ability and to detect potential overfitting, a stratified 10-fold cross-validation was used, repeated over ten cycles. The dataset was divided into ten folds: in each iteration, the model was trained on nine folds and validated on the tenth. Then the average performance accompanied is computed.

The use of the stratified technique was due to the imbalance in the outcome (23% of subjects at low risk). This approach ensures that each fold retains the original class proportions, neutralizing biases arising from the asymmetric distribution of the data that might otherwise compromise the accuracy of the classifier [26].

## 3. Results

Among the 217 participants, the majority were Italians, representing 89.8% of the sample. Europeans accounted for 4.2%, while participants of African, Asian, and Central/South American origins represented globally 6% of respondents. The study observed a relatively balanced gender distribution, with males comprising 53.5% and females 46.5%. Regarding marital status, 51.4% were unmarried, 21.8% were cohabitating, and 19.9% were married. Employment status varied significantly, with 51.9% classified as ‘other,’ followed by healthcare professionals (15.3%) and students (12.0%). Regarding educational attainment, 45.4% of respondents held an undergraduate degree, while 25.9% had completed postgraduate education. The age distribution included different age groups, with the 25–30 age group being the most represented (Table 1).

**Table 1:** Demographic and professional characteristics of travelers recruited at the USMAF-SASN clinic (N = 217).

Variables	N	%
Nationality (n = 214)		
Italian	192	89.8%
European	9	4.2%
African	5	2.3%
Asian	5	2.3%
Central/South American	3	1.4%
Gender (n = 217)		
Male	116	53.5%
Female	101	46.5%
Marital Status (n = 216)		
Single	111	51.4%
Living together	47	21.8%
Married	43	19.9%
Divorced/Separated	14	6.4%
Widowed	1	0.5%
Employment Status (n = 216)		
Healthcare professionals	33	15.3%
Students	26	12.0%
Currently unemployed	13	6.0%
Maritime and airport workers	20	9.3%
Teachers	7	3.2%
Law enforcement/Armed forces	5	2.3%
Other	112	51.9%
Education Level (n = 217)		
Postgraduate	56	25.9%
Graduate	98	45.4%
Upper Secondary Education	50	23.1%
Lower secondary education	9	4.2%
Primary education	3	1.4%
Age Group (n = 217)		
<25	19	8.7%
25–30	57	26.3%
30–35	44	20.3%
36–45	38	17.5%
46–50	13	6.0%
>50	46	21.2%

The primary reason for travel was tourism (61.6%), followed by work-related travel (23.1%). Most participants traveled with organized groups or friends (40.2%) or with family (39.2%). Popular destinations included Africa (45.2%), Central/South America (27.1%), and Asia (22.9%). Most respondents stayed abroad for one to four weeks, with hotels or resorts being the preferred accommodation choice (48.8%). Additionally, 32.4% of participants had visited high-risk infectious disease areas in the previous five years, and 38.3% reported traveling abroad more than twice yearly (Table 2).

**Table 2:** Travel characteristics of the travelers recruited at the USMAF-SASN clinic (N = 217).

Variables	N	%
Reason for Travel (n = 216)		
Tourism	133	61.6%
Work	50	23.1%
Visit friends/family	9	4.2%
Other	12	5.6%
Religion	7	3.2%
Return to home country	1	0.5%
Study vacation	4	1.8%
Accompaniment (n = 214)		
Organized group/Friends	86	40.2%
Family	84	39.2%
None/Other	44	20.6%
Destination (n = 188)		
Africa	85	45.2%
Central/South America	51	27.1%
Asia	43	22.9%
Italy	4	2.1%
Other European countries	2	1.1%
No travel planned	3	1.6%
Length of stay (n = 203)		
Less than 1 week	17	8.4%
1 week	9	4.4%
1–2 weeks	85	41.9%
2–4 weeks	67	33.0%
More than 4 weeks	12	5.9%
More than 6 months	10	4.9%
More than 1 year	3	1.5%
Accommodation type (n = 217)		
Hotel/Resort	106	48.8%
Private house	40	18.4%
Not specified	36	16.6%
Camping	15	6.9%
Hostel	11	5.2%
More than one type	7	3.2%
Other	2	0.9%
Travel to high-risk infectious countries in the past 5 years (n = 216)		
No	146	67.6%
Yes	70	32.4%
Frequency of travels abroad (n = 216)		
More than twice a year	82	38.3%
Once or twice a year	74	34.6%
Less than once a year	47	22.0%
Never	11	5.1%

As shown in Table 3, nearly half of the participants (49.3%) received vaccinations primarily to protect themselves against infections. In comparison, 18.9% were vaccinated based on their general trust in vaccine safety and efficacy before traveling to high-risk regions. The most frequently administered vaccines were for yellow fever (47%), hepatitis A (16%), and typhoid fever (16%). The primary reasons for receiving vaccinations at the USMAF Clinic were difficulties booking vaccinations through the Local Health Authority (LHA) (63.9%) and last-minute travel plans (21.2%).

**Table 3:** Information about vaccinations and knowledge of infectious diseases among travelers recruited at the USMAF-SASN clinic (N = 217).

Variables	N	%
Reason for Vaccination (n = 227) *		
Other	1	0.4%
Otherwise, I will lose my travel document	4	1.8%
To protect those around me	7	3.1%
It is mandatory in the destination country	8	3.5%
Advice of a doctor	13	5.7%
Work obligation	16	7.0%
I am afraid of contracting infectious diseases	23	10.1%
I believe in the usefulness of vaccination before traveling to a high-risk country	43	18.9%
To protect myself from infections	112	49.3%
Type of Vaccine (n = 333) *		
Polio	6	1.8%
Hepatitis B	9	2.7%
Meningococcus	9	2.7%
Diphtheria	11	3.4%
Tetanus	34	10.2%
Hepatitis A	52	15.6%
Typhoid fever	55	16.5%
Yellow fever	157	47.1%
Main Reason for Getting Vaccinated at USMAF-SASN Clinic (n = 208)		
I did not go to the clinic for vaccinations	15	7.2%
I was unaware of the vaccinations required for this destination	16	7.7%
I booked the trip unexpectedly due to work	22	10.6%
I booked the trip last minute	22	10.6%
I was unable to book with my LHA	133	63.9%
Infectious Diseases in the destination country (n = 772) *		
West Nile	11	1.4%
Mumps	13	1.8%
Zika	16	2.1%
Polio	22	2.8%
Rabies	27	3.5%
Meningococcus	28	3.6%
Dengue	33	4.3%
Hepatitis B	61	7.9%
Malaria	86	11.1%
Diarrhea	90	11.7%
Typhus	110	14.2%
Hepatitis A	123	15.9%
Yellow fever	152	19.7%

* Note: Multiple responses were allowed for this item.

As shown in Table 4, 77.1% of respondents perceived preventive measures as highly effective. However, when assessing the risk of contracting illnesses at their travel destinations, 23.2% perceived the risk as low. Information about infectious diseases prevention, was primarily obtained from health sources (63.0%) while information on the presence and type of infectious diseases at destination was mainly obtained by non-healthcare sources.

The multivariable analysis (Table 5) showed that males were significantly more likely to underestimate the pre-travel risk than females (aOR 3.63, 95% CI 1.37–9.61). Teachers demonstrated an even stronger inclination to perceive low risk, with an OR of 9.82 (95% CI 1.35–71.62), indicating that they were nearly ten times more likely to see a lower risk of contracting infectious diseases. In contrast, healthcare professionals were less likely to view low risk (aOR 0.11, 95% CI 0.01–0.97). Travelers staying in hotels or resorts were approximately six times more likely to perceive low risk than those choosing other accommodation types (aOR = 6.27, 95% CI 2.43–16.15). Furthermore, access to preventive health information from reliable sources was associated with a significantly lower odds of having low risk perception (aOR 0.20, 0.07–0.53). Conversely, travel frequency and awareness of preventive measures, did not significantly influence risk perception.

The model demonstrated satisfactory discriminatory performance with an AUC of 0.785 in cross-validation. Although this represents a decrease compared to the training (AUC = 0.848), the difference of 0.063 falls within acceptable parameters.

## 4. Discussion

This study, conducted at Rome-Fiumicino Leonardo da Vinci International Airport, provides post-pandemic evidence on infectious disease risk perception and preventive behaviors among international travelers in a real-world airport health service center.

First of all, it is important to consider how the global epidemiological landscape may have influenced participants’ perception of risk during the data collection period (from June 2023 to January 2024). Although in May 2023 the WHO declared the end of the global public health emergency for COVID-19, several concurrent outbreaks sustained public attention on infectious disease risk: respiratory illness surges in northern China, influenza A and MERS-CoV variants in the Middle East, major dengue epidemics in the Americas and Bangladesh, Marburg virus cases in Equatorial Guinea and Tanzania, the Mpox alert in the Democratic Republic of the Congo, legionellosis outbreaks in Poland, and avian influenza variants in the United Kingdom [27].

This convergence of crises during the post-pandemic transition may have influenced participants’ risk perception in opposing ways: fostering greater awareness of preventive measures on one hand, while generating information fatigue on the other [28]. The study findings should therefore be interpreted within this heterogeneous epidemiological backdrop.

In our sample, the majority of respondents reported using airport health hubs rather than local health units (LHUs) due to difficulty booking appointments at the latter. To contextualize this result, it is important to consider how travel medicine is organized in Italy. The system relies on a network of Ministry of Health-authorized International Vaccination Centers, which provide personalized pre-travel consultations structured around three pillars: updating routine vaccinations, administering internationally mandatory or destination-specific vaccines (e.g., yellow fever, hepatitis A, typhoid, dengue), and prescribing pharmacological chemoprophylaxis where indicated [29,30]. In Lazio, these services are available at both specialized LHU centers and USMAF-SANS offices [31].

This structural fragmentation has led to a documented lack of coordination and integration between territorial services and specialized care settings [32,33]. Strengthening appointment accessibility and partnerships between LHUs and travel health services is therefore essential to reducing last-minute reliance on departure-point vaccination [34,35].

Furthermore, nearly a quarter of respondents underestimated the risk of contracting diseases at their destination. These two phenomena, delayed access to services and insufficient risk awareness, may be interrelated: travelers who underestimate destination-related risks are also more likely to defer vaccination until the point of departure, at which stage immunological protection may be incomplete or, in some cases, regulatorily invalid. For example, yellow fever vaccine (the most common in this sample) requires at least ten days to confer immunity, after which the International Certificate of Vaccination or Prophylaxis (ICVP) becomes legally valid [34]. Vaccines requiring multiple doses present an even greater challenge: among travelers presenting within seven days of departure, at least one vaccine was deferred in 18% of cases due to insufficient time [35]. Both the CDC and WHO recommend initiating pre-travel consultation at least four to six weeks before departure [36,37]. Public health messaging should therefore explicitly discourage reliance on airport clinics as substitutes for timely, complete pre-travel health planning, and should emphasize the importance of anticipating travel health needs well in advance of embarkation [36].

Understanding how travelers acquire risk awareness is essential to interpreting these patterns. This divergence likely reflects a two-stage information-seeking process: non-healthcare sources such as embassies, consulates, and travel portals are consulted during the early planning phase to map the general risk landscape, alongside logistical and contextual information. Once risk awareness is established, travelers shift toward institutional healthcare sources for technical guidance on prophylaxis and preventive action. Pre-travel health services should acknowledge this dynamic and consider intervening earlier in the information journey, for instance, by partnering with travel agencies and embassy networks to embed health messaging at the initial planning stage, before travelers have already formed their risk appraisal.

The logistic regression analysis identified several individual-level predictors of risk perception, which further clarify the population groups most in need of targeted intervention. Male gender was associated with lower perceived health risk, consistent with the broader literature on gender differences in risky behavior [38,39] and with observations from the COVID-19 pandemic [40,41], where men demonstrated lower risk perception and reduced responsiveness to health warnings [23,41,42,43,44,45]. This association is particularly relevant because lower risk perception in male travelers may translate directly into reduced uptake of pre-travel vaccination, delayed health-seeking behavior, and lower adherence to chemoprophylaxis, all behaviors with measurable consequences for infectious disease risk during travel. This pattern is consistent with the previous literature, which has consistently shown that male travelers are less likely to seek pre-travel consultations [23,42]. However, further research is needed to clarify the extent to which sex-based differences in risk perception mediate these behavioral disparities, and to identify targeted interventions capable of improving preventive health engagement in this population. These findings support the need for gender-sensitive communication strategies in travel medicine. Risk communication materials and consultation approaches may need to be personalized to address the specific psychological and behavioral profiles of male travelers. Accommodation type also emerged as a significant predictor. One plausible explanation is that visible hygiene protocols in hotels and resorts may foster a sense of security, which in turn reduces travelers’ attentiveness to personal preventive behaviors [43,44]. This in in line with previous literature, hotel hygiene and safety practices act as a strong moderator between guests’ perceived travel risk and their intention to stay, highlighting the fundamental role of visible sanitation measures in shaping individual booking decisions [45]. However, this study did not specifically measure perceptions of hygiene or confidence in accommodation safety; this remains an interpretive hypothesis requiring further investigation. Health promotion interventions targeting international travelers should not assume that hotel or resort guests are a low-priority group because their inflated sense of security may render them selectively vulnerable to risks that require active individual mitigation. For example, hospitality operators should consider integrating explicit traveler-directed risk communication into the hotel environment itself or partnerships with travel medicine services. Healthcare professionals showed greater risk awareness, consistent with their medical background. Among other occupational categories, teachers showed a statistically significant association with lower risk perception; however, this result must be interpreted with caution given the small subgroup size (n = 7) and the wide confidence interval, which indicates substantial estimation instability. From a public health perspective, these findings suggest that risk communication strategies may need to be tailored to occupational context, with particular attention to non-healthcare workers who may underestimate personal risk. Finally, access to institutional healthcare sources was associated with a lower probability of perceiving low risk, underscoring the protective role of authoritative health information. Reliance on non-healthcare sources was associated with reduced adherence to preventive behaviors, consistent with vaccine hesitancy research [46] and with an Italian study showing that travelers who received direct advice from healthcare professionals were better prepared for their trip [7]. This may be linked to the fact that informal and social media channels have a fundamental role in spread health misinformation and fake news, which have been shown to erode trust in authoritative institutions and reduce adherence to recommended preventive measures [47]. These findings reinforce the importance of strengthening institutional communication channels within pre-travel health services, and of designing outreach strategies that are sensitive to gender, occupational background, and the specific information pathways that different traveler profiles are most likely to follow.

Although this study attempts to highlight some aspects of perceived health risks by travelers, there are several limitations. First, there is a potential selection bias since the recruitment of participants was done only from within the USMAF-SASN clinic; people who go to an airport clinic are more likely to be health-conscious than the general traveling population, potentially underreporting health risk perceptions or overreporting preventive behaviors. Moreover, self-reported data might be contaminated by social desirability bias. Participants might have underreported their own risk perceptions or overreported protective measures in order not to deviate from what is ideal according to behavioral models.

A further limitation lies in the cross-sectional nature of the research, which does not allow for the identification of causal relationships. Furthermore, the research was conducted at a single airport hub (Rome-Fiumicino Airport) and involved primarily Italian travelers, which limits external validity. Results may not be entirely applicable to more diverse populations or to different travel situations. Another limitation is the use of broad, non-disaggregated categories (e.g., “other” employment status), whose internal composition is unknown and may hide important differences among underlying subgroups. Finally, some occupational categories included in the analysis had very few observations; caution is advised when interpreting estimates related to these subgroups. The multivariate model included several demographic and social factors but unconsidered variables like psychological risk-taking propensity or previous experiences of illness while traveling may have played a role in the results. Future studies should use broader samples across multiple settings for better understanding health risk perceptions among international travelers.

## 5. Conclusions

This study explores key determinants of infectious disease risk perception among international travelers.

Our findings reveal that 23.2% of travelers maintain a low perceived risk of contracting infectious diseases at their destination. The multivariable regression analysis identified predictors of this low-risk perception: being a male (aOR 3.63), staying in hotels or resorts (aOR 6.27), and being a teacher (aOR 9.82). Conversely, healthcare professionals (aOR 0.11) and those who utilized healthcare-based information sources (aOR 0.20) were significantly less likely to have a low-risk perception.

These results highlight a gap in health literacy, where reliance on non-healthcare sources correlates with a failure to recognize travel-related hazards. Furthermore, the data suggest that structural barriers to accessing Local Health Units (LHUs), such as limited availability or complex booking pathways, hinder the uptake of preventive measures.

Travel medicine must move beyond traditional office-based models. Airport-based health services represent a strategic, yet under-utilized, point of contact to intercept high-risk groups who might otherwise bypass conventional healthcare pathways. By integrating airport services with local health networks, public health authorities can reduce structural barriers, provide evidence-based risk communication, and enhance global health security through improved traveler preparedness.

However, future research should assess targeted health literacy interventions and communication strategies among various traveler populations and contexts to further improve vaccination adherence and global travel health security.

## Figures and Tables

**Table 4:** Risk perception and sources of information among travelers recruited at the USMAF-SASN clinic (N = 217).

Variables	N	%
Perceived usefulness of preventive measures (0–10) * (n = 209)		
Low usefulness (0–3)	3	1.4%
Medium usefulness(4–7)	44	21.5%
High usefulness (8–10)	162	77.1%
Perception of risk of contracting infectious diseases at destination (0–10) (n = 207)		
Low risk (0–3)	48	23.2%
Medium risk (4–7)	128	61.8%
High risk (8–10)	31	15.0%
Source of information: measures to prevent disease risk ** (n = 208)		
Healthcare sources	131	63.0%
Non healthcare sources	77	37.0%
Source of information: infectious diseases at destination *** (n = 209)		
Non healthcare sources	136	65.07%
Healthcare sources	73	34.93%

* How would you assess your risk of contracting an infectious disease while traveling? 0: No risk; 10 very high” ** healthcare sources (family physicians or pediatricians) and non-healthcare sources (travel agencies, family and friends) *** healthcare sources (physicians) and non-healthcare sources (travel agencies, tour guide, online, friends, relatives, embassy).

**Table 5:** Results of the multivariable logistic regression to identify predictors of low-risk perception *.

Outcome: Low Risk Perception	aOR	95% CI	*p*-Value
Male	3.63	1.37–9.61	0.009
Employment status (Ref. Other)			
Unemployed	1.13	0.215–5.948	0.885
Teacher	9.82	1.348–71.624	0.024
Law enforcement/Armed forces	4.78	0.601–38.073	0.139
Airport worker or seafarer	1.18	0.297–4.701	0.813
Healthcare professional	0.11	0.013–0.97	0.047
Student	0.65	0.146–2.863	0.566
Information on preventive measures (Ref: Non-healthcare sources) Healthcare sources	0.20	0.072–0.537	0.001
Travel frequency: less than once a year	0.69	0.214–2.205	0.528
Accommodation type (Ref: other): Hotel/Resort	6.27	2.433–16.147	<0.001
Preventive measures awareness	1.07	0.363–3.149	0.903

* Low risk perception was defined as a score of ≤3 on a 0–10 Likert scale, in response to the question: “How would you rate the risk of contracting an infectious disease during your trip?” (where 0 indicates “No risk” and 10 indicates “Very high risk”).

## Data Availability

The data presented in this study are available on reasonable request from the corresponding author via email. The data are not publicly available due to privacy and ethical restrictions.
